# A Methodological Review of Simulation Studies Published in Pharmacoepidemiology and Drug Safety

**DOI:** 10.1002/pds.70329

**Published:** 2026-01-16

**Authors:** Ryan Muddiman, Florencia Inés Aiello Battan, John Tazare, Anna Schultze, Fiona Boland, Teresa Perez, Li Wei, Mary E. Walsh, Frank Moriarty

**Affiliations:** ^1^ School of Pharmacy and Biomolecular Sciences RCSI University of Medicine and Health Sciences Dublin Ireland; ^2^ Facultad de Estudios Estadísticos Universidad Complutense de Madrid Madrid Spain; ^3^ Department of Medical Statistics London School of Hygiene & Tropical Medicine London UK; ^4^ Department of Non‐Communicable Disease Epidemiology London School of Hygiene & Tropical Medicine London UK; ^5^ Data Science Centre, School of Population Health RCSI University of Medicine and Health Sciences Dublin Ireland; ^6^ School of Pharmacy University College London London UK

**Keywords:** review of simulations, simulation, simulation studies

## Abstract

**Purpose:**

Simulation studies are used in pharmacoepidemiology for evaluating statistical methods in a controlled setting, whereby a known data‐generating mechanism allows evaluation of the performance of different approaches and assumptions. This study aimed to review simulation studies performed in pharmacoepidemiology.

**Methods:**

We conducted a review of all papers published in the journal of Pharmacoepidemiology and Drug Safety (PDS) over the period 2017–2024. We extracted data on study characteristics and key simulation choices such as the type of data‐generating mechanism used, inferential methods tested and simulation size.

**Results:**

Among 42 simulation studies included, 34 (81%) were informing comparative effectiveness/safety studies. Twenty‐two studies (52%) used simulation in the context of a clinical condition, and 36 (86%) used Monte‐Carlo simulation. Inputs not derived from empirical data alone (*n* = 22, 52%) or in combination with real‐world data sources (*n* = 19, 45%) were most often used for data generation. The complexity of simulations was often relatively low: although 31 studies (74%) generated data based on other covariates, time‐dependent covariates (*n* = 3) and effects (*n* = 4) were rarely implemented. Bias was the most often used performance measure (*n* = 26, 62%), although notably 18 studies (43%) did not report uncertainty in the method.

**Conclusion:**

Simulations contributed a relatively small number of articles (3.2% of 1320) to PDS over 2017–2024. Greater focus on evaluating methods and inferential approaches, using simulation studies that are appropriately complex given clinical realities, may be beneficial to the pharmacoepidemiology field.

## Key Points

- Simulation studies comprised 3.2% (*n* = 42 of 1320) of articles reported in Pharmacoepidemiology and Drug Safety over 2017–2024. The type of simulations was mostly Monte‐Carlo‐based, where probability distributions were used to produce outcomes and/or covariates for analysis that have desired conditional or marginal properties.
- 55% of studies were based on a specific clinical context that informed choices of the causal structure or inputs to the simulation.
- Studies rarely simulated time‐varying effects (*n* = 4) or covariates (*n* = 3), which may have implications for the transportability of the study's conclusions.
- The most common performance metric was bias (*n* = 26), but uncertainty was often not reported (*n* = 18).

## Plain Language Summary

Simulation studies are tools that enable the performance evaluation of one or more statistical methods. Simulations often include several choices of parameters in their design and our paper aimed to summarise the specific practices in use within pharmacoepidemiology. This study reviewed all articles published in the journal Pharmacoepidemiology and Drug Safety over the period 2017–2024 which included simulation to test a statistical method. We found 42 such papers and described their key details with a summary of results. Studies typically used fully synthetic data generation and reported bias in the method using the true value of the estimate. Fifty‐five percent of studies incorporated knowledge of a drug or disease in the generation of the data to more closely match reality.

## 1 Introduction

Simulation studies are empirical experiments that typically generate data from pseudo‐random sampling, apply a quantitative method and evaluate the method [1]. They are particularly useful when data complexity makes theoretical evaluation of methods impossible [2], such as estimating the power of a test given the data structure alone. This is particularly true in observational pharmacoepidemiology datasets where complex and dynamic processes are involved [3]. Simulations can evaluate methods of statistical inference such as estimation or hypothesis testing in a controlled setting, where aspects of the underlying data‐generating mechanism are controlled. In pharmacoepidemiological research, this can be applied to the estimands of interest, most often relative drug efficacy/safety or the prevalence of clinical outcomes.

Various approaches can be used in the DGM. Where real‐world data (RWD) is available, the DGM can involve plasmode simulation [4], which samples covariates from a real dataset and simulates outcomes, enhancing external validity. Monte‐Carlo simulations [5, 6] instead involve repeating simulations while varying input parameters to provide generalised results across scenarios. The approaches for simulation studies commonly used by the pharmacoepidemiology community have not been explored to date. Understanding current trends in the field could support our understanding of current best practice and highlight potential future areas for improvement. Therefore, this study aimed to review simulation studies and approaches used in pharmacoepidemiology in a single journal.

## 2 Methods

The protocol for this review was pre‐registered [7] and the details are summarised below.

### 2.1 Search

Our study builds on a previous review of code‐sharing practices in pharmacoepidemiology and was restricted to articles published in the journal Pharmacoepidemiology & Drug Safety (PDS) [8]. We included simulation studies identified in this previous review (2017–2022) and augmented them with our own comparable search of the literature from January 2023 to December 2024. Thus, our study covers the period 2017–2024. PubMed was searched using the easyPubMed package [9] in R [10] (see the Supporting Information for the search string). Our finalised search was conducted on 17/01/2025.

### 2.2 Eligibility

Articles included were original research articles or brief reports where computer‐simulated data was used to evaluate a statistical inference method using a known DGM or known estimand/target of inference. Commentaries and review articles were excluded. Studies that did not involve computer simulations (to avoid including pedagogical healthcare simulations) or did not quantitatively measure the absolute or comparative performance of an inferential method (e.g., health economic models) were also excluded. Reasons for full‐text exclusion are given in the Supporting Information.

### 2.3 Screening

Covidence was used for screening of titles/abstracts of simulation studies from the previous review, and records from the new search, by two independent reviewers (R.M., F.I.A.B.), with conflicts resolved by a third reviewer (F.M.). Full texts were reviewed by the same two independent reviewers, with conflicts and excluded records checked and resolved by the same third reviewer.

### 2.4 Data Extraction

Data extraction was performed by one reviewer and a second reviewer also extracted 20% of articles to assess consistency. In addition to publication metadata from the easyPubMed package, data was manually extracted using an MS Excel spreadsheet with data validation based on the protocol [7]. The type of data extracted was classified using the Aims, Data‐generating mechanism, Estimand, Method, Performance measures (ADEMP) framework by Morris et al. [1]. Where data items were not reported in the article, code (if provided) was inspected to identify missing items.

### 2.5 Data Analysis

We used R (version 4.4.1) to analyse extracted data, focusing on descriptive statistics. The code and data are on Zenodo [11].

## 3 Results

Forty‐two studies were included (see Supporting Information for the PRISMA [12] flow chart). Table 1 summarises the characteristics of included articles.

**TABLE 1:** Summary of characteristics of included simulation studies (*n* = 42).

Variable	Category^a^	*n*	%
Study type	Comparative effectiveness/safety	34	81%
	Signal detection	4	10%
	Exposure measurement	2	5%
	Covariate balance	1	2%
	Outcome rate estimation	1	2%
Aspect of study simulation informed	Analysis	29	69%
	Design	9	21%
	Design and analysis	4	10%
Type of intervention examined	Not applicable	19	45%
	Drug	12	29%
	Vaccine	4	10%
	No specific intervention	4	10%
	Multiple	2	5%
	Surgery	1	2%
Drug class or vaccine	Not applicable	26	62%
	Other	6	14%
	Statin	3	7%
	Vaccine	3	7%
	Anti‐cancer	2	5%
	Anticoagulant	2	5%
Clinical condition examined^b^	No/Not applicable	20	48%
	Diseases of the circulatory system	7	17%
	Neoplasms	5	12%
	Endocrine, nutritional and metabolic diseases	3	7%
	Diseases of the respiratory system	1	2%
	Codes for special purposes	1	2%
	Diseases of the nervous system	1	2%
	Pregnancy, childbirth and the puerperium	1	2%
	Diseases of the digestive system	1	2%
	Diseases of the musculoskeletal system and connective tissue	1	2%
	Factors influencing health status and contact with health services	1	2%
Year	2017	4	10%
	2018	3	7%
	2019	10	24%
	2020	6	14%
	2021	3	7%
	2022	6	14%
	2023	5	12%
	2024	5	12%
Simulation type	Monte Carlo	36	86%
	Plasmode	5	12%
	Bootstrap^c^	1	2%
Data generation mechanism^d^	Random sampling	36	86%
	Stochastic process	6	14%
Time parameterisation	None	23	55%
	Continuous	15	36%
	Discrete	3	7%
	Not reported	1	2%
Source for data generation	Synthetic data	22	52%
	Both	19	45%
	Real world data	1	2%
Factor variables^e^	Covariate magnitude	24	57%
	Effect magnitude	18	43%
	Data Generating Mechanism structure	12	29%
	Not applicable	6	14%
Quantity of interest	Cumulative risk	21	50%
	Instantaneous risk	12	29%
	Other	9	21%
Primary inferential method categorised^f^	Nonparametric	17	40%
	Maximum likelihood	9	21%
	Partial likelihood	11	26%
	Other	4	10%
	NA	1	2%
Performance measure for assessing inferential method	Bias	26	62%
	Other	10	24%
	Power	3	7%
	None	3	7%
Uncertainty analysis	Summary dispersion	19	45%
	None	18	43%
	Coverage^g^	4	10%
	Other	1	2%
Programming language	R	20	48%
	Unknown	9	21%
	Stata	5	12%
	R, & SAS	4	10%
	SAS	3	7%
	MATLAB	1	2%
Published code	No	24	57%
	Yes	18	43%
Component of code shared	Not applicable	24	57%
	Data generating mechanism and analysis	11	26%
	Analysis only	5	12%
	Data Generating Mechanism only	2	5%
Location of shared code	NA	22	52%
	Supporting Information	13	31%
	GitHub	2	5%
	Code package	2	5%
	Package	1	2%
	GitHub, Supporting Information	1	2%
	GitLab	1	2%

^a^ Further definitions of the categories are in the Supporting Information.

^b^ The condition was classified according to the ICD‐10 chapter.

^c^ This study utilised full bootstrapping to obtain the data, whereas plasmodes contain covariate sampling with simulated outcomes.

^d^ We classified a DGM as using random sampling when using time‐independent outcome generation and as a stochastic process where they generated a random variable indexed by a separate time variable.

^e^ Factors are variables that are systematically varied through repeated simulations. This does not sum to the number of studies since more than one variable may be factored.

^f^ Methods were grouped as nonparametric estimation (where the method of moments or non‐likelihood estimation was used e.g., Kaplan–Meier estimators), maximum likelihood estimation (under a specified distributional model), partial likelihood estimation (e.g., proportional‐hazards models) or other (e.g., classification/clustering).

^g^ Coverage is a performance measure relating to correct interval estimation and as such is classed here within uncertainty analysis.

**FIGURE 1:**
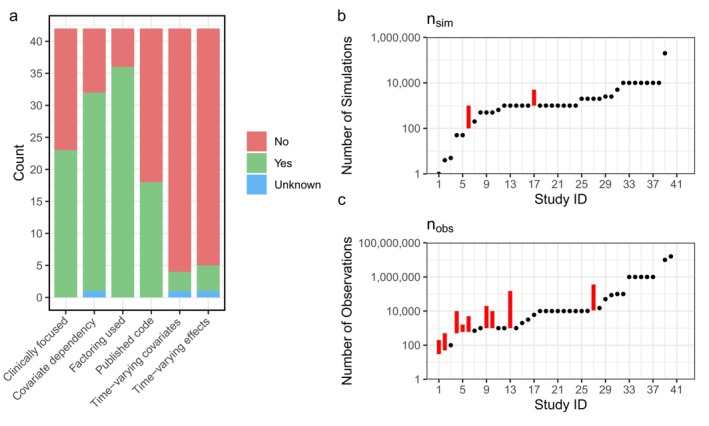
(a) Bar chart of binary study characteristics, (b) Distribution of values used across studies for the number of simulations (b) and number of observations (c). Study ID is not equal between (b) and (c). (Red bar is a range).

The results of binary parameters are shown in Figure 1a. Twenty‐three studies (55%) focused on a specific clinical question, with diseases of the circulatory system most commonly studied (17%). Most simulations aimed to inform comparative effectiveness/safety studies (81%) and specifically their analysis (69%). Where medical interventions were named, drugs were the most common type (29%).

Simulations consisted of Monte‐Carlo type (88%), plasmodes (12%) and bootstrap (2%). The DGM was characterised as random sampling in 36 studies (86%) and by a stochastic process in the remaining studies. Fifteen studies (36%) had time defined continuously, and three (7%) used discrete time. DGM parameters were most frequently synthetic (52%) with other studies using RWD alone or a mix of both.

Thirty‐one studies (74%) generated data that had exposure or outcomes dependent on other covariates, and 36 studies (86%) factored one or more variables in the simulation. The most commonly factored parameters were the magnitude of covariates (57%). Three studies (7%) included time‐varying covariates and four (10%) included time‐varying effects in simulations, although time was not a relevant aspect of some studies. We show the distribution (over all studies) of the range of the number of simulations (*n*_sim_) and number of observations (*n*_obs_) per simulation in Figure 1b,c, respectively. The number of simulations was mostly a single value (88%), while the number of observations varied more frequently (24%).

The most common target was risk measures (50%) such as odds ratios or relative risks; other measures such as hazard ratios were less common (29%). Nonparametric estimation was the most common method used (40%). Twenty‐six studies used bias as the primary measure of performance (62%), and uncertainty was mostly reported using dispersion measures over the simulations such as confidence intervals and standard deviations (45%). The most common programming language was solely R (48%); however, nine studies (21%) did not indicate the software used. Code was shared in less than half of studies (43%). The shared code was primarily used for both the data generation mechanism and analysis (26%) but some code only performed one of these functions. The shared code was in the Supporting Information of 13 studies (31%).

## 4 Discussion

Simulation studies represented a very small number of articles in PDS over 2017–2024. The ADEMP framework [1] can aid reporting of simulation studies. We did not aim to formally quantify adherence to ADEMP, but as the framework guided our data extraction, we can infer aspects of reporting quality.

### 4.1 Aims and Methods

Most studies clearly stated their simulation aims and the analytical methods used. Nineteen studies referenced a medical intervention in the aim or conceptual design. This can align the DGM to expected biological mechanisms and will lead to the simulation being relevant to applied researchers (for 45% of studies, an intervention was not applicable). Methods using nonparametric inference were most common. This may be because, in comparative effectiveness studies, the canonical analysis is group comparison using the Kaplan–Meier estimator for a failure time and various hypothesis tests for non‐failure time outcomes. Nonparametric bootstrapping is also robust and simple to implement, making it popular for estimating incidence in effectiveness studies. Therefore, the methods implemented represent the standard statistical approaches in pharmacoepidemiology.

Over half of studies focused on a clinical condition, allowing potential DGM inputs to be narrowed to those expected in reality. In such cases, relative risks or other causal or associational measures may be based on real data or expert judgement. When the simulation is not based on a clinical scenario, the choice of input distributions may be less clear and may result in selections that are unrealistic in practice. Comparing the simulated dataset to RWD using summary statistics is a potential solution.

### 4.2 Estimands

Estimands were mostly those used for comparative effectiveness. While 50% of studies targeted risk measures such as odds ratios or relative risks, the theoretical specification of these quantities and their link to the data‐generating mechanism was rarely made explicit.

### 4.3 DGM

The description of DGMs was detailed but often lacked justification of assumptions. Studies mostly used pseudo‐random sampling for outcome generation within a Monte‐Carlo simulation framework incorporating synthetic covariates. Some studies lacked reporting of key simulation details such as the software used and choice of time definition in the DGM. Despite minimal legal/regulatory impediments, code was often not shared. This means that code cannot be independently verified. It also prevents reuse, such as extending a DGM to other use‐cases or comparative testing of novel methods using the same DGM. Even where the DGM is fully described in a paper, this creates additional barriers and workload to conduct replications. Code sharing may be rare due to the potential for added scrutiny of external researchers and low perceived gain to those sharing.

The lack of a clear aim and/or reasons for choices made in the DGM can lead to biased conclusions. The performance measures are empirical data that are highly dependent on the DGM, which means simulation conclusions should account for the inevitably different DGM operating in reality. Furthermore, the choice of methods and their precise implementation, particularly in comparison studies, can be difficult to justify due to design bias, i.e., knowledge of the DGM may affect the choice of methods. Prespecifying all methods, using a blinded analyst with knowledge of output data only, could resolve such issues.

Plasmodes were uncommon, potentially due to the lack of access to RWD which requires ethical approval and also lack of guidance for implementation. Simulating from parametric distributions is much easier from a practical standpoint. Alternatively, published RWD effect estimates can be used in a Monte‐Carlo simulation with synthetic covariates, although typically only the summary statistics of covariate distributions are published. The number of observations is restricted to the available data in a plasmode. In Monte‐Carlo studies, the number of observations is determined by the researchers and thus varied substantially, with a median of 10 000 for included studies. This parameter is effectively an aspect of the DGM and should match the sample size for the intended population to which the methods will apply.

Conversely, the number of simulations is not bounded by any external data. While a higher value of *n*_sim_ will reduce the sampling error of Monte‐Carlo estimates, there is typically no justification given for the actual choice of *n*_sim_, and in practice, this often relates to available computing resources. Therefore, it is recommended to include *n*_obs_ as a factor, while *n*_sim_ only needs to be sufficiently large to ensure acceptable precision in the performance measures. The drastic range of *n*_sim_ across studies (10^6^ to 1) may be indicative of the various types of estimands of interest. In general, simulation studies should estimate the optimal number of repetitions depending on the required precision of the performance measures, as suggested by Burton et al. [6].

Time‐varying effects and covariates were simulated in four and three studies respectively. The choice of time‐constant parameters means that the simulated data represented a static risk/relative risk of outcomes over time, which may not represent reality in some scenarios [13]. Not all statistical inference requires a consideration of time‐dependent variables, but given that 29% of studies targeted an instantaneous risk, their findings may be limited to the time‐constant case. Insufficient DGM complexity will lead to performance measures that do not apply to real data. A conservative approach is to implement rigorous data complexity (such as non‐homogenous processes, non‐proportional hazards, heteroskedasticity) by default unless there is evidence suggesting otherwise.

### 4.4 Performance Measures

Performance measures were frequently reported without quantifying uncertainty (*n* = 18, 43%), meaning the sampling variation due to the finite number of simulations cannot be ascertained. Since simulation conclusions are reliant on the performance measures, the possibility of sign‐reversal in estimates or false negatives/positives should always be considered using the Monte‐Carlo standard error.

While some studies did evaluate multiple statistical methods and reported different performance measures, it was previously observed that the relative performance of different statistical methods depends on the DGM structure and the type of distributions used [14] and at a minimum, simulations should incorporate both time‐constant and time‐varying effects, and dependence on past events. Thus, DGMs were often not adequately varied or suitably complex for the conclusions of many studies to be applicable to observational datasets.

While this review was limited to a single journal, it suggests low numbers of simulation studies published within the pharmacoepidemiology community. Our journal restriction means that the simulations reviewed may not represent the entirety of the field and are specific to the authors of PDS. Future research including simulation studies in journals with a purely statistical or methodological focus would likely have more generalisable results at the expense of specificity to pharmacoepidemiology. Greater focus on evaluating methods and inferential approaches, using simulation studies that are appropriately complex given clinical realities, may be beneficial to the field.

## Funding

This work was supported by the Wellcome Trust (DIAMOND programme Career Development Award, grant number 227348/Z/23/Z) and the Spanish Ministry of Science, Innovation and Universities (PID2022‐137050NB‐I00).

## Ethics Statement

This study used publicly available data and did not require ethical approval.

## Conflicts of Interest

A.S. is employed by LSHTM on a fellowship funded by GSK.

## Supporting information


**Data S1:** Supporting Information.
